# Emotional but not physical maltreatment is independently related to psychopathology in subjects with various degrees of social anxiety: a web-based internet survey

**DOI:** 10.1186/1471-244X-12-49

**Published:** 2012-05-25

**Authors:** Benjamin Iffland, Lisa M Sansen, Claudia Catani, Frank Neuner

**Affiliations:** 1 Department of Psychology, Bielefeld University, Postbox 100131, , 33501, Bielefeld, Germany; 2 Christoph-Dornier-Stiftung für Klinische Psychologie, Bielefeld, Germany

**Keywords:** Child abuse, Child neglect, Abuse, Neglect, Social anxiety disorder, Anxiety

## Abstract

**Background:**

Previous studies reported that social phobia is associated with a history of child maltreatment. However, most of these studies focused on physical and sexual maltreatment whilst little is known about the specific impact of emotional abuse and neglect on social anxiety. We examined the association between emotional maltreatment, including parental emotional maltreatment as well as emotional peer victimization, and social anxiety symptoms in subjects with various degrees of social anxiety.

**Methods:**

The study was conducted as a web-based Internet survey of participants (*N* = 995) who had social anxiety symptoms falling within the high range, and including many respondents who had scores in the clinical range. The assessment included measures of child maltreatment, emotional peer victimization, social anxiety symptoms and general psychopathology.

**Results:**

Regression and mediation analyses revealed that parental emotional maltreatment and emotional peer victimization were independently related to social anxiety and mediated the impact of physical and sexual maltreatment. Subjects with a history of childhood emotional maltreatment showed higher rates of psychopathology than subjects with a history of physical maltreatment.

**Conclusions:**

Although our findings are limited by the use of an Internet survey and retrospective self-report measures, data indicated that social anxiety symptoms are mainly predicted by emotional rather than physical or sexual types of victimization.

## Background

Child maltreatment refers to various forms of abusive and potentially harmful parenting that threatens the healthy development of a child. Several retrospective as well as prospective studies have found that child maltreatment is associated with psychopathology, in particular affective and anxiety disorders as well as substance abuse [1-5]. Most of this research refers to the consequences of physical abuse, sexual abuse, physical neglect or combinations of these types of maltreatment.

However, abusive treatment that does not involve any physical transgression by a caregiver but is characterized instead by acts such as verbal hostility, taunting, belittling, rejection as well as emotionally neglectful parenting that is unavailable, detached, avoidant and unresponsive to the child’s needs and desires [6] is also maltreatment. These types of abuse have been called emotional maltreatment [7], which can be subdivided into emotional abuse and neglect. Although this denomination may be considered somewhat unfortunate (since any type of abuse has an emotional impact), it is well established in the scientific literature.

In addition to the emotional maltreatment that involves abusive treatment by parents, non-physical types of victimization (also referred to as emotional victimization) are highly common among peers and may also be associated with psychopathology in the victims [8]. Emotional peer victimization involves bullying, verbal threats or aggression, malicious manipulation of a relationship, friendship withdrawal and damaging another’s peer relationships [9].

While some studies have indicated that the outcome of emotional maltreatment may be as severe as the consequences of physical or sexual maltreatment [6,10], research on the specific impact of emotional maltreatment on psychopathology is still scarce. However, there is some evidence for an association of a history of emotional abuse and neglect by caretakers and anxiety disorders, particularly social phobia [11-15]. In addition, peer victimization seems to be associated with clinical social phobia [16] and characteristics of sub-clinical social anxiety, such as fear of negative evaluation, social avoidance of general and new situations, as well as physiological symptoms in social situations [9,17-19].

However, former reports of the association between maltreatment and social anxiety were limited by the fact that they did not control for different kinds of maltreatment. Previous studies focused either on physical or sexual abuse and neglect, emotional maltreatment by caretakers or emotional peer victimization. As different types of abuse and neglect are highly inter-correlated and co-occur in most populations [20-23], it is important to study the unique effect of single types of maltreatment. In this study, we planned to resolve this contamination of types of child abuse. We aimed to explore the specific impact of emotional types of ill-treatment, including emotional abuse and neglect within the family as well as emotional peer victimization. Using regression analyses as well as mediation tests we aimed to disentangle the independent contribution of emotional forms of maltreatment while controlling for physical types of abuse in a sample of subjects with high levels of social anxiety. We hypothesized that, while all types of abuse are associated with psychopathology in this sample, the effects of physical maltreatment are to a large extent mediated by emotional maltreatment. Furthermore, we postulated an independent effect of emotional types of peer victimization. Large sample sizes as well as a large variance of both predictor and outcome variables were therefore required. We used a web-based Internet survey posted on self-help web pages for anxiety patients to recruit subjects and assess data. We expected that this recruitment would allow us to study a large sample of subjects scoring highly for social anxiety.

## Methods

### Participants

The survey was advertised through various popular German language web pages and newsgroups that attract high numbers of anxious subjects (e.g. http://www.panik-attacken.de, http://www.verrueckt.de, http://www.schuechterne.org). These advertisements were linked to a study web page that included the survey. Initially, 1691 individuals accessed this web site. Out of these, 658 did not provide full data as they logged off prematurely. Their data was excluded from further analysis. In addition, data from individuals who reported that they were under the age of 18 were excluded from analyses. Full data for 995 subjects was available. The demographic characteristics of this group are presented in Table 1.

**Table 1:** **Subject characteristics and mean values on psychopathology (*N*** **= 995)**

	
Age, *M* (*SD*)	31.32. (10.44)
Gender, % male (*n*)	29.60 (295)
Nationality, % german (*n*)	91.56 (911)
Family status, % single (*n*)	45.10 (449)
Educational level, % graduation and higher (*n*)	65.23 (649)
Employment, % unemployed (*n*)	14.07 (140)
Psychotherapeutic treatment, % lifetime (*n*)	59.30 (590)
Medication, % psychopharmacological treatment (*n*)	27.70 (276)
Social Phobia Scale, *M* (*SD*)	31.45 (19.80)
Social Interaction Anxiety Scale, *M* (*SD*)	39.70 (20.91)
Brief Symptom Inventory - Global Severity Index, *M* (*SD*)	1.37 (.83)
Childhood Trauma Questionnaire, *M* (*SD*)	46.01 (16.95)
Emotional Abuse, *M* (*SD*)	11.39 (5.32)
Emotional Neglect, *M* (*SD*)	13.15 (5.57)
Physical Abuse, *M* (*SD*)	6.88 (3.48)
Physical Neglect, *M* (*SD*)	8.27 (3.40)
Sexual Abuse, *M* (*SD*)	6.31 (3.46)
Peer victimization, *M* (*SD*)	14.60 (9.09)

### Procedure

The study web page was implemented using Enterprise Feedback Suite (EFS) Survey (Globalpark, 2007). Data was collected over an 18 month period. On entering the web page, participants met a welcome page with an introductory statement and study information. Subjects were informed that participation was voluntary, that data was collected anonymously and that they could discontinue at any time. They were invited to fill in a socio-demographic questionnaire as well as the study questionnaires (see below). Finally, participants had the chance to comment on the study and ask questions of the researchers. The study was approved by the Ethical Committee of the Department of Psychology of Bielefeld University.

### Instruments

#### Social anxiety symptoms

For the assessment of social phobia, the German version of the Social Phobia Scale/Social Interaction Anxiety Scale (SPS/SIAS) [24] was used. The SPS was developed to assess anxiety related specifically to social performance, whereas the SIAS was designed to measure anxiety related to general social interaction. Both, the SPS and the SIAS consist of 20 items using a five-point Likert scale that are rated from 0 (not at all) to 4 (extremely) indicating how characteristic or true the statements are for the respondent. On both scales total scores range from 0 to 80. Cut-off scores of 20 on the SPS and 30 on the SIAS indicate a clinical relevant level of social anxiety [25]. The German version of the SPS/SIAS has shown high levels of internal consistency and convergent, but deficient discriminant validity [24].

#### Child maltreatment

The German version of the Childhood Trauma Questionnaire (CTQ) [23] was used to assess different types of childhood maltreatment. The 28-item self-rated scale distinguishes five areas of maltreatment (sexual abuse, emotional neglect, emotional abuse, physical neglect and physical abuse). The items are rated from 1 (never true) to 5 (very often true) with a possible range of subscale scores of 5 to 25. The psychometric properties of the German version were similar to the original version and it has been shown to be a reliable and valid screen for childhood maltreatment. Internal consistency of all scales except physical neglect was shown to be high (Cronbach´s *α* > .89). Correlations with self-reported measures for posttraumatic stress, dissociation and general psychopathology were low to moderate [23]. In the present study, Cronbach´s alpha was *α* = .87 for all items. Apart from physical neglect (Cronbach´s *α* = .64), internal consistency of all scales was high (Cronbach´s *α* > .87).

#### General psychopathology

In order to measure psychopathology and psychological distress, the German version of the Brief Symptom Inventory (BSI) [26-28] was used. The BSI is a 53-item short form of the Symptom Check List 90 (SCL-90). It produces the same nine primary symptom dimensions (somatization, obsessive-compulsity, interpersonal sensitivity, depression, anxiety, hostility, phobic anxiety, paranoid ideation and psychoticism). Furthermore, three global indices measure general psychological distress. These include the Global Severity Index (GSI), the Positive Symptom Total (PST) and the Positive Symptom Distress Index (PSDI). Each item is rated on a 5-point Likert scale ranging from 0 (not at all) to 4 (extremely) and is considered to be rated for the experience of the past 7 days including today.

#### Emotional peer victimization

To assess emotional peer victimization, we used a checklist that was recently developed and validated in our working group by Sansen et al. [29]. The Fragebogen belastender Sozialerfahrungen (FBS; questionnaire of stressful social experiences) assesses various forms of childhood maltreatment occurring amongst peers. It consists of a list of 22 stressful social situations (i.e. “I was excluded from games or activities by other children or adolescents”, “I have been laughed at in the presence of other children”). For each situation respondents are asked whether or not they have experienced this situation during childhood (age of 6 to 12) or adolescence (age of 13 to 18). Each item is rated binary for the two age-groups separately. The total score ranges from 0 to 44. The total-score showed a satisfying stability over a period of twenty months (*r* = .89). Construct validity has been confirmed through correlations with measures of psychological symptom distress and social anxiety. Moderate correlations with the scales of the CTQ indicate that the FBS assesses an additional construct of child maltreatment. Hence, use of the FBS allows estimation of the cumulative effects of varying kinds of child maltreatment. For this study, the sum score of both scales was used as indicator of emotional peer victimization during development.

#### Statistical analyses

All statistical analyses were carried out using the Statistical Package for the Social Sciences SPSS 19. We applied cut off scores established by Walker [30] for the CTQ subscales to decide whether the dichotomous criteria of different types of child abuse were fulfilled. Maltreatment is assumed when threshold scores for emotional abuse (10), emotional neglect (15), physical abuse (8), physical neglect (8) and sexual abuse (8) are met. Pearson correlations were calculated to examine the association of childhood maltreatment to social anxiety symptoms as well as psychopathology. In the second step, we utilized linear regression models to examine the specific association of different types of childhood maltreatment and psychopathology. For this purpose, we calculated composite scores for emotional maltreatment (sum-score of the CTQ subscales emotional neglect and emotional abuse) as well as physical maltreatment (sum score of physical neglect and physical abuse). In the third step, we tested the hypothesis that the effects of physical maltreatment are partly mediated by emotional maltreatment using mediation analysis [31]. To estimate the effect-sizes and to test significance of the indirect effect, we used the Sobel test as well as a nonparametric approach using 10.000 bootstraps [31].

## Results

Subjects were 995 individuals, predominantly women (*n* = 700, 70,4%) recruited through the Internet. The average age was *M* = 31.32 (*SD* = 10.44). Table 1 presents participants’ means on the assessments.

On the SPS 65.6% (*n* = 653) of the subjects reached the cut-off score for clinical relevance and 64.6% (*n* = 643) scored higher than the cut-off on the SIAS. The subjects’ mean on the Global Severity Index of the BSI was significant higher than the norm score of adult non-patients (*M* = .31, *SD* = .23; *t* (1593) = 30.579, *p* < .001).

We found 703 subjects (70.76%) meeting Walker’s threshold severity criteria for at least one type of childhood abuse or neglect, as measured by the CTQ subscales. Threshold levels were met for emotional abuse for 54.6% (*n* = 543), emotional neglect for 39.7% (*n* = 395), physical abuse for 22.4% (*n* = 223), physical neglect for 48.6% (*n* = 484) and sexual abuse for 15.1% (*n* = 150). There were 16.0% (*n* = 159) meeting threshold levels for two subtypes, 14.2% (*n* = 141) met threshold levels for three subtypes, 13.0% (*n* = 129) for four subtypes, and 6.6% (*n* = 66) for all five subtypes.

In the analysis of the relationship between the different types of maltreatment and social phobia and psychopathology, all correlations were found to be significant (see Table 2). However, the correlations between the emotional types of maltreatment and social phobia and psychopathology were found to be higher.

**Table 2:** Correlations between different types of maltreatment and Social Phobia and Psychopathology

	**SPS**	**SIAS**	**BSI**
	*r*	*r*	*r*
Peer victimization	.39***	.44***	.47***
Emotional Abuse	.42***	.37***	.46***
Emotional Neglect	.41***	.42***	.44***
Physical Abuse	.23***	.18***	.28***
Physical Neglect	.30***	.25***	.33***
Sexual Abuse	.16***	.10**	.20***

* *p* < .05, ** *p* < .01, *** *p* < .001. SPS, Social Phobia Scale; SIAS, Social Interaction Anxiety Scale; BSI, Brief Symptom Inventory - Global Severity Index.

The impact of emotional and physical maltreatment on social phobia was examined in gender- and age-adjusted regression analyses (see Table 3). The emotional maltreatment sum-score and emotional peer victimization were associated with the SPS (Full model: *F* (5, 989) = 65.50; adjusted R2 = .245; *p* < .001) and the SIAS (Full model: *F* (5, 989) = 72.77; adjusted R2 = .265; *p* < .001), whereas the physical maltreatment sum-score did not achieve significance on the SPS. Furthermore, we found a significant association of the emotional maltreatment score and emotional peer victimization with psychopathology (Full model: *F* (5, 988) = 87.49; adjusted R2 = .303; *p* < .001). The physical maltreatment score and psychopathology were not associated.

**Table 3:** The association of different types of maltreatment and social phobia and psychopathology

		**SPS**			**SIAS**			**BSI**	
	***r***	**β**	***P***	***r***	**β**	***P***	***r***	**β**	***P***
Emotional peer victimization	.22*	.23*	.000	.30*	.31*	.000	.31*	.32*	.000
Emotional Maltreatment	.29*	.41*	.000	.29*	.41*	.000	.27*	.36*	.000
Physical Maltreatment	-.06	-.08	.068	-.12*	-.16*	.000	-.03	-.04	.303

All Regression models are adjusted for age and gender. * *p* < .001. SPS, Social Phobia Scale; SIAS, Social Interaction Anxiety Scale; BSI, Brief Symptom Inventory - Global Severity Index.

**Figure 1:**
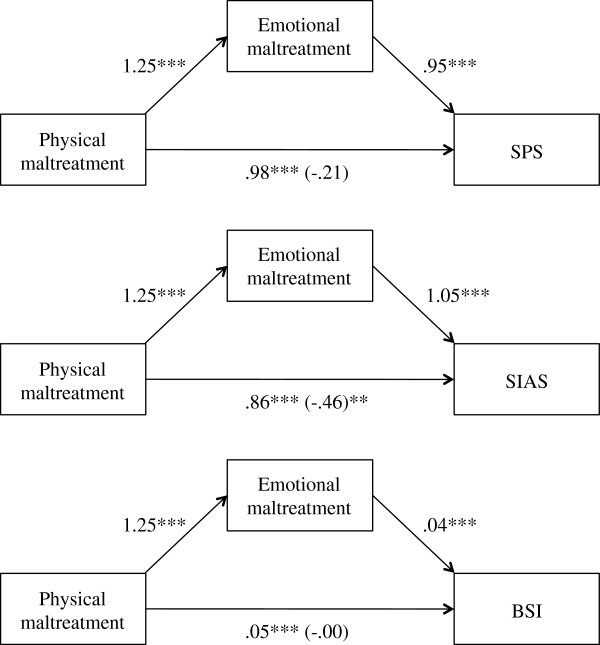
Standardized regression coefficients for the relationship between physical maltreatment and psychopathology as mediated by emotional maltreatment. The standardized regression coefficients between physical maltreatment and psychopathology controlling for emotional maltreatment are in parentheses. * p < .05, ** p < .01, *** p < .001, two-tailed. SPS, Social Phobia Scale; SIAS, Social Interaction Anxiety Scale; BSI, Brief Symptom Inventory - Global Severity Index.

Without controlling for emotional maltreatment, physical maltreatment is significantly associated to the SPS, the SIAS and psychopathology (see Figure 1). To investigate whether the association of physical maltreatment with the SPS is mediated by emotional maltreatment we conducted a test for simple mediation [31], with the physical maltreatment score as independent variable, the emotional maltreatment score as mediator, and the SPS as dependent variable (see Figure 1). When the emotional maltreatment score was added as mediator, the association of the physical maltreatment score with the SPS was eliminated, this reduction was significant (*Z* = 11.00; *p* < .001). In a second mediation analysis we tested whether the relationship between physical maltreatment and the SIAS is mediated by emotional maltreatment. The relationship was partially and significantly reduced when adding emotional maltreatment as a mediator (*Z* = 11.42; *p* < .001). We then investigated whether the emotional maltreatment score mediates the association of the physical maltreatment score with psychopathology. A test for simple mediation showed that the association of physical maltreatment with psychopathology was completely and significantly reduced after adding a mediator (*Z* = 11.17; *p* < .001). Effect-sizes and significances of the indirect effects are presented in Table 4.

**Table 4:** Effect-sizes and significances of the indirect effects

**IV**	**MV**	**DV**	**Effect**	***Z* score**	** Bootstrap results**	
					** IV ➔ MV ➔ sDV**	
					**Effect**	**SE**
Physical Maltreatment	Emotional maltreatment	SPS	1.19	11.00***	1.19	.11
Physical Maltreatment	Emotional maltreatment	SIAS	1.32	11.42***	1.33	.11
Physical Maltreatment	Emotional maltreatment	BSI	.05	11.17***	.05	.01

IV, independent variable; MV, mediator variable; DV, dependent variable; SPS, Social Phobia Scale; SIAS, Social Interaction Anxiety Scale; BSI, Brief Symptom Inventory - Global Severity Index, * *p* < .05, ** *p* < .01, *** *p* < .001, two tailed.

**Figure 2:**
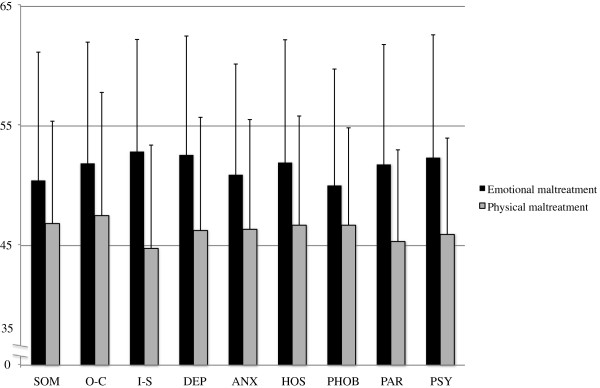
**Normalized standard means (*t*-scores) for the comparison of subjects who suffered emotional maltreatment and subjects who suffered physical maltreatment on the BSI subscales. SOM, somatization; O-C, obsessive-compulsity; I-S, interpersonal sensitivity; DEP, depression; ANX, anxiety; HOS, hostility; PHOB, phobic anxiety; PAR, paranoid ideation; PSY, psychoticism.**

To analyze whether emotional maltreatment leads to a qualitatively different profile of psychopathology than physical maltreatment we divided the whole sample into two groups. The first group consisted of subjects who suffered either emotional neglect or emotional abuse but no kind of physical or sexual maltreatment. Those subjects who experienced physical abuse or neglect but no emotional or sexual maltreatment were assigned to the second group. Walker’s threshold severity criteria were used to allocate the subjects. The groups’ profiles on the subscales of the BSI show that there were no specific symptom profiles for emotional versus physical maltreatment. Rather, we found that subjects who suffered emotional maltreatment showed significant higher scores on almost all BSI subscales. With the exception of the BSI subscale somatization (*t* (237) = 2.86, *p* = .005) all t-tests adjusted for multiple testing were significant (all *p*’s < .004; see Figure 2). In addition, subjects with a history of emotional maltreatment had significantly higher scores on the Global Severity Index of the BSI (*t* (237) = 5.17, *p* < .001) and the SPS/SIAS (SPS: *t* (237) = 4.91, *p* < .001; SIAS: *t* (237) = 4.39, *p* < .001).

## Discussion

In a sample of subjects with a high range of social anxiety symptoms, we found that reports of emotional maltreatment were more directly and strongly associated with psychopathology than physical types of maltreatment. The analyses showed that the association of physical maltreatment and social anxiety was significantly mediated by emotional maltreatment. As well as emotional maltreatment by caretakers, emotional types of peer victimization seem to be an additional unique predictor of social anxiety symptoms. Compared to subjects who exclusively report a history of physical maltreatment, subjects reporting exclusively emotional maltreatment showed higher rates on all types of broad-band psychopathology.

Consistent with prior research, our data showed an association of childhood maltreatment with psychopathology, and in particular, social anxiety symptoms. Previous studies focused on the impact of physical and sexual abuse [32-35] and postulated that these types of maltreatment are a risk factor for mental disorders including social phobia [36,37]. In contrast, our study found only a small independent relationship between sexual and physical maltreatment and social anxiety, while regression analyses revealed that emotional maltreatment was independently associated to social phobia. The impact of physical maltreatment on social anxiety disappeared when emotional maltreatment was controlled for, suggesting that emotional abuse and neglect has a greater role than physical maltreatment in the development of socially anxious symptoms. These findings are consistent with recent findings of Acarturk et al. [32]. In a population based prospective study on the incidence of social anxiety disorder they found that childhood emotional neglect was a significant predictor of social anxiety disorder. Other studies found greater symptom severity of social anxiety disorder in patients who experienced emotional maltreatment [12,13].

In our study, emotional maltreatment mediated the association of physical maltreatment with social anxiety and psychopathology. Similar effects have been documented in various populations before. For example, in a study on the psychological health of battered women, Tomasulo [38] documented that non-physical abuse mediated the relationship of physical abuse and psychological distress as well as interpersonal functioning. Congruent with these findings, Lewis et al. [39] showed that emotional abuse was uniquely associated to depression, above and beyond the effects of physical abuse.

Second to child maltreatment, reports of emotional peer victimization were independently related to social anxiety symptoms in our sample. This is consistent with previous studies that indicated that poor peer relations, including peer rejection and neglect, were related to social anxiety disorder [8,19,40]. Socially anxious adolescents showed decreased rates of peer acceptance and increased rates of peer- and self-reported victimization [41].

Besides the impact of emotional maltreatment on social anxiety, our data suggested influences of emotional maltreatment on various types of psychopathology. Previous studies reported a prominent role of emotional maltreatment in predicting eating disorders, depressive symptoms and risky health behaviors [11,42,43]. Wright et al. [10] found high rates of dissociative, anxious and depressive symptomatology in a sample of college students who reported emotional abuse and neglect. In line with these findings, subjects reporting exclusively emotional maltreatment in this study were found to have higher rates on almost all subscales of the BSI. Hence, emotional maltreatment rather than physical maltreatment is suggested to have negative implications on most domains of psychopathology. These results are congruent with the findings of Gauthier et al. [44], who reported greater problems in multiple domains of psychopathology in women reporting a history of emotional neglect.

By studying the relative effect of various kinds of child maltreatment, the present study addressed limitations of previous studies that have examined the impact of child maltreatment on social anxiety. By subdividing the context of the emotional maltreatment to relational and peer victimization, it was possible to identify the cumulative and divergent effects of the type and context of maltreatment. Our findings indicate that the relative impact of emotional forms of abuse and neglect may be higher than indicated by the previous research on child maltreatment which had mainly focused on physical and sexual types of abuse and neglect. One reason for the reluctance to study emotional abuse could be that reports of physical abuse may be more salient and alarming, thereby hiding the impact of seemingly more subtle forms of abuse [39]. In addition, the conceptualization and definition of emotional maltreatment is difficult [40], as it refers to a harmful range within a continuum of neglectful and verbally abusive behavior.

The present study has several limitations. Recruitment through the Internet was advantageous in that it meant that it was possible to collect data from large sample with a broad range of maltreatment as well as symptoms. However, the generalizability of our findings is limited. Although data of the clinical scales indicate that we have probably examined a large proportion of subjects who fulfill the criteria for social phobia and other mental disorders, we cannot confirm the clinical status on an individual level as no clinical interviews were carried out. It would be important to validate our findings in smaller samples of patients with social anxiety disorder. In addition, the generalizability of our findings is limited by the high rate of female participants. Population-based studies showed that social anxiety disorder is more prevalent among women [45-47]. However, in clinical samples prevalence rates were found to be equal for both sexes. For further studies, a more appropriate sex ratio would be desirable. There have been some reservations about the use of internet recruitment and assessment [48]. For example, we cannot be certain that participants gave accurate personal information. However, face-to-face interviews may not be an advantage in this regard, as the responses might be more strongly biased by social desirability. Another problem with collecting data over the Internet is that the possibility that single subjects have participated more than once cannot be ruled out. However, it does not seem likely that a significant number of individuals would make this effort in view of the high availability of competing sites on the Internet [48]. Selection factors are unpredictable in Internet recruitment and as a result it is only possible to use a convenience sample in such studies. However, we did not aim to make any statements about prevalence rates in the general population. It is improbable, though not impossible, that our findings about the independent association of emotional maltreatment to social anxiety were influenced by selection factors. Finally, assessment of childhood maltreatment in this study is based on retrospective accounts and self-report, which is subject to recall biases. Particularly socially anxious subjects may be more likely to overestimate the occurrence of negative social situations as measured by CTQ and FBS. However, analyses of the validity of retrospective reports generally concluded that biases are present but not sufficiently large enough to invalidate retrospective studies [49]. Under-reporting rather than over-reporting was found in retrospective assessment of childhood maltreatment. As our cross-sectional design does not allow causal conclusions, the interpretation of the data remains tentative. Besides being a predictor of social anxiety, findings of Siegel et al. [9] suggested that emotional maltreatment may also be a consequence of social anxiety. To clarify causality in the association of childhood emotional maltreatment and social anxiety, further research using longitudinal prospective designs is desirable.

## Conclusion

In conclusion, our findings suggest that social anxiety symptoms are mainly predicted by emotional types of victimization, either in childhood through the caretakers, or during childhood and adolescence through the peer. In contrast to previous studies focusing on physical and sexual abuse, this finding indicates that more awareness should be paid to emotional forms of abuse and neglect during development to prevent psychological disorders and to identify children at risk.

## Competing interests

The authors declare that they have no competing interests.

## Authors’ contributions

BI participated in the conception and design of the study, collected data, performed the statistical analyses and interpretation of findings, and drafted the manuscript. LMS participated in the conception and design of the study, collected data, performed the statistical analyses and interpretation of findings, and helped to draft the manuscript. CC participated in the conception and design of the study, made substantial contributions to the interpretation of findings and revised the manuscript. FN participated in the conception and design of the study, made substantial contributions to the statistical analyses and interpretation of findings, helped to draft and revised the manuscript. All authors read and approved the final manuscript.

## Pre-publication history

The pre-publication history for this paper can be accessed here:

http://www.biomedcentral.com/1471-244X/12/49/prepub
